# Editorial: Cancer therapy: The challenge of handling a double-edged sword

**DOI:** 10.3389/fphar.2022.1007762

**Published:** 2022-09-09

**Authors:** Kulmira Nurgali, John A. Rudd, Halina Was, Raquel Abalo

**Affiliations:** ^1^ Institute for Health and Sport, Victoria University, Melbourne, VIC, Australia; ^2^ Department of Medicine Western Health, University of Melbourne, Melbourne, VIC, Australia; ^3^ Regenerative Medicine and Stem Cells Program, Australian Institute for Musculoskeletal Science (AIMSS), Melbourne, VIC, Australia; ^4^ School of Biomedical Sciences, The Chinese University of Hong Kong, Shatin, Hong Kong SAR, China; ^5^ The Laboratory Animal Services Centre, The Chinese University of Hong Kong, Shatin, Hong Kong SAR, China; ^6^ Laboratory of Molecular Oncology and Innovative Therapies, Military Institute of Medicine, Warsaw, Poland; ^7^ Área de Farmacología y Nutrición, Departamento de Ciencias Básicas de la Salud, Universidad Rey Juan Carlos (URJC), Alcorcón, Spain; ^8^ Unidad Asociada I+D+i del Instituto de Química Médica (IQM), Consejo Superior de Investigaciones Científicas (CSIC), Madrid, Spain; ^9^ High Performance Research Group in Physiopathology and Pharmacology of the Digestive System (NeuGut-URJC), URJC, Alcorcón, Spain; ^10^ Grupo de Trabajo de Ciencias Básicas en Dolor y Analgesia de la Sociedad Española del Dolor, Madrid, Spain; ^11^ Grupo de Trabajo de Cannabinoides de la Sociedad Española del Dolor, Madrid, Spain

**Keywords:** cancer, chemotherapy, immunotherapy, side effects, sequelae

Cancer is a worldwide public health priority with a high socioeconomic burden. Unfortunately, cancer treatments often lead to side effects and impairments, greatly feared by patients and clinicians, which need to be identified, properly managed, and prevented. However, this is often not achieved, thus reducing the patient’s quality of life (QoL) and survivorship. As a follow-up to our previous Frontiers Research Topic (FRT) (Nurgali et al., 2018), this new FRT deals with the knowledge gaps about the side effects of anti-cancer therapy, as well as the strategies aimed at improving QoL during and after cancer treatment.

Chemotherapy-induced neurotoxicity is an important problem that may be long-lasting, often irreversible, and may severely affect the QoL of cancer survivors. As shown by Was et al. (2022) in their review, platinum-based agents, taxanes, vinca alkaloids, proteasome inhibitors, thalidomide analogs and other chemotherapeutic drugs may cause neurotoxic effects in both the peripheral and central nervous systems, including neuropathic pain, chemobrain, enteric neuropathy, as well as nausea and emesis. Direct neurotoxicity is considerable on peripheral nerves (including the enteric nervous system) and the area postrema (which is not protected by the blood-brain barrier) but is more limited to a few lipid-soluble drugs in the central nervous system. However, indirect mechanisms, mainly due to inflammatory reactions, are increasingly recognized to contribute to chemotherapy-induced neurotoxicity, including the central nervous system toxicity that causes cognitive impairment known as “chemobrain” or “chemofog”. Cannabinoids, due to their demonstrated neuroprotective role in other cognitive-related neuropathologies, were suggested as a potential therapeutic tool against chemotherapy-induced cognitive side effects (Boullon et al., 2021).

Peripheral neuropathy is a disabling side effect with few effective preventive strategies. Leen et al. (2022) performed a systematic review and meta-analysis, which focused on the effectiveness of neuroprotectants for paclitaxel-induced peripheral neuropathy. Interestingly, non-pharmacological interventions (mainly cryotherapy, compression therapy, and massage therapy) seemed to be more effective than pharmacological interventions (gabapentin, omega-3 fatty acids, vitamin E and N-acetylcysteine) in the 14 controlled trials that were eligible for the study. However, the results may be biased by the small sample size, methodological heterogeneity, inconsistencies in reporting across the studies, and evidence considered low to moderate.

Kerckhove et al. (2021), using cluster analysis, showed that oxaliplatin-treated colorectal cancer patients might be subclassified according to the severity of peripheral neuropathy and the proportion of neuropathic pain. Similarly, Selvy et al. showed that bortezomib (a proteasome inhibitor) induced sensory peripheral neuropathy in approximately 25% of the studied multiple myeloma patients, which was associated with considerable psychological distress (anxiety, depression, insomnia) and decreased QoL. Remarkably, in both studies, duloxetine, the only treatment recommended by the American Society of Clinical Oncology for chemotherapy-induced peripheral neuropathy, was not used, leading to suboptimal management of patients. Thus, more personalized and effective management, in line with the current guidelines, is needed.

Immune checkpoint inhibitors (ICI) are among the newest therapies against cancer and are generally considered safer for patients than conventional chemotherapy. However, they may induce relevant immune-related adverse events (IRAEs), of which dermatologic adverse events, particularly pruritus and rash, are the most common and worrying, since they may lead to drug withdrawal and decreased QoL. From an analysis of 50 randomized clinical trials, Ge et al. found that Programmed cell death protein 1 (PD-1)/Programmed cell death-ligand 1 (PD-L1) inhibitors displayed a better safety profile than Cytotoxic T lymphocyte associate protein 4 (CTLA-4) inhibitors, in both monotherapy and combined immunotherapy regimens. Furthermore, the addition of chemotherapy did not seem to increase skin toxicity. Despite some heterogeneity of the studies included in this systematic review and meta-analysis, the results obtained seem to be robust and highlight the need to study ICI-induced dermatologic adverse events more deeply to improve the management of patients receiving such treatments.

It has been shown that therapeutic outcomes are better in patients that develop skin issues during ICI therapy. However, this is not true for every patient. Li et al. reported the case of a patient with cervical cancer who developed a severe bullous skin reaction after treatment with anti-PD-1 monoclonal antibodies (sintilimab and toripalimab), eventually leading to death, probably associated with infection and sepsis (Li et al.). Skin toxicity may also be induced by other cancer treatments. Shu and Zheng reported the case of a patient with metastatic colorectal cancer who developed severe rashes after 2 weeks of treatment with fruquintinib, a highly selective inhibitor of vascular endothelial growth factor receptor.

Gastrointestinal and cardiovascular toxicities are common side effects of anti-cancer treatment. For the gastrointestinal side effects, the mechanisms of nausea and emesis were examined from a central and peripheral perspective in the context of cancer treatment with platinum-based therapy (Was et al., 2022). The contribution of G protein-coupled receptor (GPCR) downstream signaling in general emetic circuitry was introduced with emphasis on the role of phospholipase C (PLC) underpinning the emetic action of a broad range of chemotherapeutics, including those activating 5-HT_3_, tachykinin NK_1_, dopamine D_2/3_ receptors, which are well-known targets for established anti-emetic drugs. The PLC inhibitor, U73122, had broad inhibitory action against these challenges. Notwithstanding these important observations, the emetic action of a PLC activator, m-3M3FSB, was antagonized by palonosetron and netupitant, highlighting the complexity of GPCR (and ligand-gated ion channel receptors) in the emetic reflex (Zhong & Darmani, 2021). Inhibiting PLC in brain areas/tissues outside the emetic reflex may be expected to result in a wide range of side effects and problems for the cancer patient as GPCRs that are coupled to PLC are important to a number of physiological systems. However, as palonosetron and netupitant both did not completely protect against m-3M3FSB, it may mean there is an unidentified GPCR coupled to PLC that exists distal to 5-HT_3_ and NK_1_ receptors in the emetic reflex, which may represent a new key target for anti-emetic drug development.

The emetic action of cisplatin in the periphery was shown to be more complex than first understood, with regional differences in gastrointestinal acute toxicity being revealed using extracellular recording techniques. The discovery that cisplatin could disrupt slow waves in a tissue-specific manner is a new finding that could be relevant to nausea and emesis, reduced appetite, and changes in gastrointestinal motility. How cisplatin induces changes at the cellular level is presently not fully understood but it may be related to the cell types and/or their density in each region (Tu et al., 2021). Could the understanding of the regional mechanisms of cisplatin-induced slow wave dysrhythmia also lead to a new range of therapeutics to reduce side effects? Certainly, cisplatin caused changes in gastric slow wave rhythm during the acute and delayed phases of emesis, respiratory changes, mild hypothermia, and reduced blood pressure, indicating the extent of toxicity involving multiple systems (Tu et al., 2021).

ICIs have modernized cancer chemotherapy, with a notably lower potential to cause nausea and emesis, but the potential to cause exaggerated IRAEs, particularly in the gastrointestinal system should not be overlooked. Colitis, hepatobiliary and pancreatic disorders can present when using PD-1 (e.g. pembrolizumab, nivolumab) and PD-L1 (e.g. avelumab) inhibitors and CTLA-4 ligands (e.g. ipilimumab). Whilst the incidence of adverse effects may be considered low (1–30%, depending on the agent and organ system), the impact is a rapid deterioration of function and increased risk of mortality (Bai X et al., 2021). Understanding the risk factors with each compound class is necessary to minimize these risks, particularly considering the increasing trend towards polytherapy.

Chemotherapy-induced cardiotoxicity was also reviewed from the perspective of cardiac function (ejection volume, blood pressure) and endothelial toxicity in preclinical and clinical studies. Cisplatin is known to have cardiotoxic properties relating to alterations of autonomic output and direct organ action (Tu et al., 2021). Vincristine is able to cause dysfunction of the aorta, mesenteric vascular smooth muscle, and endothelial health, through inducing the expression of tumor necrosis factor-α and inducible forms of nitric oxide synthase; these effects were not permanent and would recover back to normal after treatment cessation Herradon et al. Trastuzumab, an antibody targeting human epidermal growth factor receptor 2 (HER2) used in the treatment of breast cancer, increases the risk of cardiovascular disease and death from cardiovascular disease. A meta-analysis approach was used to investigate if common cardiovascular medications including angiotensin receptor blockers, angiotensin-converting enzyme inhibitors, and beta-blockers may have protective effects. However, none of the treatments were effective (Li et al., 2021a). This may be because the mechanism and role of HER2 are intracellular and involved in oxidative stress in cardiomyocytes, which is not impacted by medications that protect against events via modulation of blood pressure. Atezolizumab (a PD-1 antibody) and bevacizumab (an endothelial growth factor antibody targeting angiogenesis) cause hypertension when used alone and in combination for cancer treatment. The significance of the impact of hypertension was placed into perspective when considering the advantages of combining atezolizumab and bevacizumab for cancer treatment (Jiang et al., 2021).

Pneumonitis is an uncommon but very serious side effect of chemo and immunotherapy often resulting in significant clinical deterioration, acute respiratory failure accounting for a high percentage of fatalities, and pulmonary fibrosis resulting in chronic respiratory failure. In most cases, treatment-induced pneumonitis occurs within days, weeks, or months following the start of anti-cancer treatment. Rapoport et al. (2021) report that treatment with PD-1 and PD-L1 inhibitors increases the risk of pneumonitis five to six fold compared to cytotoxic chemotherapy. The onset of immunotherapy-induced pneumonitis, indicated by dyspnea, dry cough, low-grade fever, and chest pain, varies widely from 1.9 to 24 months (median 2.8 months). Clinical cases of interstitial pneumonitis associated with the epidermal growth factor receptor tyrosine kinase inhibitor, afatinib, in patients with advanced non-small cell lung cancer and with taxane-based chemotherapy (paclitaxel) in breast cancer patients have been analyzed by Liu et al. and (Ardolino et al., 2021). The authors concluded that patients presenting with a fever, dyspnea, and shortness of breath should be assessed early for drug-induced interstitial pneumonitis. Thoracic imaging and bronchoscopy should be performed to exclude an atypical infection. High doses of glucocorticoid therapy initiated promptly when drug-induced interstitial pneumonitis is suspected, can increase patients’ survival rate.

Chemotherapy-induced myelosuppression is another common adverse effect of anti-cancer treatment. It manifests as reduced counts of white blood cells due to the inhibition of their differentiation from bone marrow hematopoietic stem cells (BMHSCs). Current drugs promoting the recovery of BMHSC functions, such as erythropoietin and thrombopoietin, are expensive, while a recombinant colony-stimulating factor can potentially stimulate tumor cell proliferation. Gu et al., (2021) tested the anti-myelosuppressive efficacy of the main bioactive components of traditional Chinese medicine Shuanghuang Shengbai granule in a mouse model of cyclophosphamide (CTX)-treated lung adenocarcinoma. The study demonstrated that these bioactive components improve the hematopoietic function of BMHSCs via upregulation of high mobility group box 1 protein through inhibition of miR-142-3p, which alleviates CTX-induced cell injury and myelosuppression in this model.

As mentioned above, immunotherapy is one of the most promising emerging anti-cancer therapies. However, immunotherapies may cause several adverse effects, including the so-called hyperprogressive disease (HPD) associated with the accelerated proliferation of cancer cells, the rapid progression of the disease, and a poor prognosis for the patient. Shen et al. focused in their comprehensive review on defining HPD characteristics and prognostic biomarkers, potential HPD mechanisms, and avoidance strategies after ICI treatment. Another less obvious side effect of the therapy might be cancer relapse and the acquisition of resistance to further treatment. Olszewska et al. (2021) suggested that cellular senescence/senescence escape may be one of the mechanisms of this phenomenon. Authors showed that hypoxia, which is a common feature in tumors, may enhance the escape of cancer cells from therapy-induced senescence. This resistance can be overcome by co-administration of the antimalarial, analgesic, anti-inflammatory, and autophagy-inhibiting agent, hydroxychloroquine. These results could be implemented in patients, although verification in preclinical models and long-term perspective is required.

Long-term adverse effects of chemotherapy, including secondary malignant neoplasms, heart, liver, bone toxicity, impaired vision, obesity, impact on fertility, and neurocognitive impairments have been evaluated in survivors of pediatric acute lymphoblastic leukemia (Al-Mahayri et al., 2021). Several genetic factors increasing the risk of long-term toxicity were identified. Some pharmacogenetic biomarkers were found to be associated with a high risk of steroid sensitivity. Identification of these biomarkers opens avenues for the recognition of patients at a higher risk and the development of mitigation strategies to prevent these long-term sequelae of chemotherapy.

The nutritional status of elderly patients undergoing chemotherapy has a strong impact on the adverse effects of chemotherapy and patients’ survival outcome. A study conducted by Chang et al. of docetaxel-treated metastatic castration-resistant prostate cancer patients revealed that poor nutrition is associated with a higher incidence of febrile neutropenia, nausea, vomiting, liver toxicity, cardiovascular events, and reduced survival. Therefore, the geriatric nutritional risk index, which evaluates serum albumin levels and the ratio of actual and ideal body weights, could be used as a simple and valuable tool in making the choice and the doses of chemotherapy.

New drugs or combinations of existing drugs are continuously designed, re-designed, and tested to increase their effectiveness and reduce adverse effects for patients. Bai Y et al. (2021) expanded their previous *in vitro* studies with the novel anti-cancer agent oxovanadium complex VO(hntdtsc) (NPIP) in an animal model. They showed that VO(hntdtsc) (NPIP) blocked the tumor growth and induced apoptosis of human cervical cancer cells in mice xenograft models. Of importance, it seemed to be less toxic than cisplatin. Tayarani-Najaran et al. in their comprehensive review described recent studies on the anticancer effects of Auraptene, a bioactive monoterpene coumarin isolated from *Citrus aurantium* and *Aegle marmelos* that belong to the Rutaceae family. Auraptene showed inhibitory and chemo-preventive effects in several cancer cell lines *in vitro* and in animal models. It also has anti-bacterial and anti-fungal activities and an excellent safety profile making it worthy for consideration in clinical trials. Wang et al. (2021) performed an analysis of 3 open-label randomized phase 2/3 clinical trials with a total of 1,108 previously untreated advanced soft tissue sarcoma patients. The aim of the study was to compare monotherapy with doxorubicin/adriamycin (ADM) alone to ADM combined with ifosfamide (AI). Analysis of parameters such as overall survival (OS), progression-free survival (PFS), and objective response rate did not show a statistically significant advantage of AI combination therapy over ADM monotherapy. Both therapies were also comparable in terms of adverse effects, as shown by discontinuation rate and toxic death. Liang et al., (2021) conducted retrospective studies on 225 patients with advanced hepatocellular carcinoma. Patients were subjected to hepatic arterial infusion chemotherapy (HAIC) using the classical chemotherapeutics combination FOLFOX (oxaliplatin, 5-fluorouracil, and leucovorin) alone or the same HAIC plus tyrosine kinase inhibitor, sorafenib. HAIC plus sorafenib significantly improved OS, PFS, and disease control rates compared with HAIC alone, although the frequencies of some adverse effects were higher in the HAIC plus sorafenib group than in the HAIC alone group. Therefore, a large-sample, prospective, randomized controlled trial is needed to verify these promising results. Chen et al. performed a detailed analysis of 463 clinical trials with 132 drugs for prostate cancer (PC) completed over the years 2010–2020. In recent years, long-acting hormone therapy, radiotherapy combined with ICIs, and gene-targeting chemotherapeutic agents have revolutionized the concept of PC treatment, prolonging patients’ lives and improving their QoL. The authors noted that only 31 clinical trials testing 19 new compounds were conducted in China at that time. Given the large number of patients, China could be an important area for expanding PC drug research.

In conclusion, the present FRT, like our previous one (Nurgali et al., 2018) has already generated a lot of interest with high numbers of views and citations. However, many aspects of this topic area were not specifically dealt with, such as the impact of cancer chemotherapy on sensory functioning (hearing, vision, taste), vital organs like the kidneys, the musculoskeletal system, and approaches to maintaining fertility during and after treatment. Given the increasing incidence, prevalence, and impact of cancer on the health system, research on cancer treatments will continue to expand, and the evaluation of their negative impacts and how to overcome them should also follow. The efforts posed by researchers in this area should help cancer treatments be better tolerated and more effective, leading to enhanced health and wellness of cancer survivors.
